# Increased Chromogranin A Cell Density in the Large Intestine of Patients with Irritable Bowel Syndrome after Receiving Dietary Guidance

**DOI:** 10.1155/2015/823897

**Published:** 2015-03-30

**Authors:** Tarek Mazzawi, Doris Gundersen, Trygve Hausken, Magdy El-Salhy

**Affiliations:** ^1^Division of Gastroenterology, Department of Medicine, Stord Hospital, 5416 Stord, Norway; ^2^Division of Gastroenterology, Department of Clinical Medicine, University of Bergen, 5020 Bergen, Norway; ^3^National Centre for Functional Gastrointestinal Disorders, Department of Medicine, Haukeland University Hospital, 5021 Bergen, Norway; ^4^Department of Research, Helse-Fonna, 5528 Haugesund, Norway

## Abstract

The large intestine contains five types of endocrine cells that regulate its functions by sensing its luminal contents and releasing specific hormones. Chromogranin A (CgA) is a common marker for the gastrointestinal endocrine cells, and it is abnormal in irritable bowel syndrome (IBS) patients. Most IBS patients relate their symptoms to certain food elements. The present study investigated the effect of dietary guidance on the total endocrine cells of the large intestine as detected by CgA in 13 IBS patients. Thirteen control subjects were also included. Each patient received three sessions of dietary guidance. Colonoscopies were performed on controls and patients (at baseline and at 3–9 months after receiving guidance). Biopsy samples from the colon and rectum were immunostained for CgA and quantified by computerized image analysis. The densities of CgA cells in the total colon (mean ± SEM) among the controls and the IBS patients before and after receiving dietary guidance were 83.3 ± 10.1, 38.6 ± 3.7, and 64.7 ± 4.2 cells/mm^2^, respectively (*P* = 0.0004), and were unchanged in the rectum. In conclusion, the increase in CgA cell density after receiving dietary guidance may reflect a change in the densities of the large intestinal endocrine cells causing an improvement in the IBS symptoms.

## 1. Introduction

Irritable bowel syndrome (IBS) is a common gastrointestinal (GI) disorder that is present in almost one-third of consultations with gastroenterologists [1, 2]. IBS predominates among females, and its reported prevalence worldwide ranges from 5% to 20% [1–6]. The symptoms of IBS vary from mild to severe and are attributed to visceral hypersensitivity, disturbed GI motility, or abnormal GI secretion [7–9]. Gastrointestinal sensation, motility, and secretion are regulated by the neuroendocrine system of the GI tract [7, 8], which comprises the GI endocrine cells and the enteric nervous system [1, 10]. The GI endocrine cells comprise almost 1% of all epithelial cells in the GI tract and are considered the largest endocrine organ in the body [11–13]. These cells project specialized microvilli into the lumen, which sense its contents (mainly nutrients) and release specific hormones into the lamina propria [14–26]. The large intestines, namely, the colon and rectum, contain five types of endocrine cell: serotonin-, peptide-YY-, somatostatin-, oxyntomodulin- (enteroglucagon-), and pancreatic-polypeptide-producing cells [27–29].

Chromogranin A (CgA) is considered to be a common marker for the GI endocrine cells [30–32]. The CgA cell density was previously found to be abnormal in the colon of patients with IBS [33] but not in the rectum [27]. Such abnormalities in the gastrointestinal cells in IBS patients, involving not only the large intestines but also the gastric mucosa, have been suggested to play a major role in the pathophysiology of IBS [7, 34–36].

Most patients with IBS relate their symptom development to the consumption of certain food elements [37, 38]. Dietary management achieved by providing individualized guidance has been reported to improve the symptoms and quality of life of IBS patients [39, 40]. The same dietary guidance normalizes the gastric endocrine cells [41, 42], and thus the present study investigated whether dietary guidance affects the total endocrine cells of the large intestine as detected by CgA.

## 2. Material and Methods

### 2.1. Patients and Controls

Patients referred to the Gastroenterology Division, Stord Hospital, Norway, who were aged 18–70 years and had IBS according to the Rome-III criteria, were recruited for this study. Women who were pregnant or lactating or who received a caesarean section or hysterectomy and patients who had organic gastrointestinal or any other systemic diseases, abused drugs, or suffered from serious psychiatric illness were excluded. Patients with previous abdominal surgery, with the exception of appendectomy, were also excluded.

Seven patients did not use medications prior to the study, while six patients consumed one or a combination of several medications. Four patients consumed proton pump inhibitors (PPI), one used an antihypertensive angiotensin II receptor antagonist, three used medications against allergies, two consumed contraceptive pills, two took thyroxin substitution tablets, one used inhalator against asthma, and two used antidepressants/anxiolytics. These patients were informed not to take any kind of PPI during the study.

Patients with gastrointestinal bleeding, where the source of bleeding was identified as hemorrhoids (*n* = 3) or angiodysplasia (*n* = 1), or healthy subjects who underwent a colonoscopy due to health worries caused by family member(s) having been diagnosed with gastrointestinal cancer (*n* = 9) were used as controls. The control group consisted of nine females and four males with a mean age of 54 years (range 26–70 years).

The study was approved by the local Committee for Medical Research Ethics West, Bergen, Norway, and was performed in accordance with the Declaration of Helsinki. The patients provided both oral and written consent to participate.

### 2.2. Study Design

A total of 46 patients were included in the study, comprising 35 females and 11 males with a mean age of 35 years (range 18–69 years). All of the patients were examined physically and had their blood tested in order to exclude the presence of inflammation, infection, and other organic diseases. Segmental biopsy samples were taken during the colonoscopies in order to exclude microscopic colitis. All of the patients received three sessions of individualized dietary guidance lasting about 45 min each. The sessions were scheduled at intervals of at least 2 weeks and were conducted by a nurse experienced in dietary guidance in IBS. The patients underwent colonoscopies, before the first dietary guidance session and at 3–9 months (median 4 months) after the third session.

### 2.3. Individualized Dietary Guidance

The information about IBS and dietary guidance was delivered both orally with the help of charts and as written illustrated information. During the first session, the patients received general information about IBS, the importance of regular and healthy eating habits, and the food elements that trigger IBS symptoms, that is, insoluble dietary fiber and poorly absorbed FODMAPs (fermentable oligosaccharides, disaccharides, monosaccharides, and polyols). The daily consumption of dairy products was allowed during the study, with the patients being informed that lactose-free milk and lactose-free dairy products do not provoke IBS symptoms. The patients were asked to test a fat-, protein-, or carbohydrate-rich/poor diet for 2 weeks. During this period the patients were instructed to register in a diary their daily intake of food and fluids along with any symptoms they experienced including the frequency and degree of abdominal pain, abdominal distension, and stool frequency and consistency. Consuming food items supplemented with probiotics, antibiotics, and other medications was not permitted during the course of the study unless otherwise specified. In the second session, the nurse briefly repeated the information given at the first session and together with the patient identified the symptom-triggering food elements based on an assessment of the patient's diary. The patients were then encouraged to change the proportions of protein, fat, and carbohydrate in their diet, avoid FODMAPs-rich items and insoluble fiber, and consume vegetables and fruits with lower amounts of FODMAPs and insoluble fiber. During the third session, the patients shared their experiences concerning the dietary guidance with the nurse. The nurse together with the patient then designed a suitable diet for the patient to strictly follow until the endpoint of the study.

### 2.4. Dietary Assessment

Dietary intake was assessed by using MoBa food frequency questionnaire (MoBa FFQ). This questionnaire is semiquantitative and self-administered and reports the frequency and meal size related to a series of foods and beverages consumed over a defined period of time. The data were then analyzed using software to calculate the nutrient content of the diet. The MoBa FFQ was developed and validated by the Norwegian Institute of Public Health in Oslo, Norway [43, 44]. This questionnaire inquires about the consumption of 225 food items and identifies the respondent's dietary habits, including the consumption of oral supplements (if any), according to typical Norwegian meal patterns. The participants completed the MoBa FFQ form before the first session and again at least 3 months after the last session of dietary guidance [39].

### 2.5. Colonoscopies, Tissue Sampling, Histopathology, and Immunohistochemistry

Colonoscopies were performed on both patients and controls. Biopsy samples were taken from the right colon (the cecum, the ascending colon, and the right half of the transverse colon), the left colon (the left half of the transverse colon, the descending colon, and the sigmoid colon), and the rectum about 15 cm from the anus. Four biopsies were taken from each segment.

The biopsy samples were fixed overnight in 4% buffered paraformaldehyde, embedded in paraffin wax, and then cut into 5 *µ*m thick sections. The sections were stained with hematoxylin-eosin and immunostained using the method involving the Avidin-Biotin complex (ABC) with the Vectastain ABC kit (Vector Laboratories, Burlingame, CA, USA) and the chromogen 3,3′-diaminobenzidine peroxidase substrate (DAB) kit (Vector Laboratories) as described in detail previously [27]. Briefly, after 2 hours of incubation with a monoclonal mouse anti-N-terminal of purified CgA primary antibody (code number M869; Dako, Glostrup, Denmark) diluted to 1 : 1000, at room temperature, the sections were washed in phosphate-buffered-saline (PBS; pH 7.4) and incubated for another 30 min at room temperature with biotinylated swine anti-mouse IgG (diluted to 1 : 200). The antibodies were raised to N-terminal of purified chromogranin A. These antibodies reacted with human chromogranin A as validated earlier [33]. The slides were then washed with PBS, incubated for a further 30 min with ABC (diluted to 1 : 100), and then submerged in DAB, followed by counterstaining with hematoxylin.

### 2.6. Computerized Image Analysis

The densities of CgA in the right colon, left colon, and rectum of patients with IBS and controls were evaluated using computer software (Cell ^∧^D, Olympus, Tokyo, Japan) under a ×40 objective. The number of CgA positive cells and the area of the epithelial cells were measured in ten randomly chosen fields. Each field represented a tissue area of 0.14 mm^2^. The CgA cell density is expressed herein as the number of cells per square millimetre of epithelium. All of the slides were quantified by the same person (Tarek Mazzawi) who was unaware of the identity of the sections.

### 2.7. Statistical Analysis

The paired* t-*test was used to compare patients before and after receiving dietary guidance and the unpaired* t-*test was used to compare between the male and the female patients before and after receiving dietary guidance. The data are presented as mean ± SEM values. *P* values < 0.05 were considered indicative of statistical significance.

## 3. Results

### 3.1. Patients and Controls

Of the 46 patients recruited in the study, 24 lost their motivation to continue participating in the study and withdrew their consent due to symptom improvement as a consequence of receiving dietary guidance and/or being unwilling for a second colonoscopy to be performed. Two patients were excluded because of noncompliance, two were diagnosed with celiac disease, one was diagnosed with lupus, and one received antibiotic treatment because of gastroenteritis. A further three patients were excluded because they became pregnant during the study and moved abroad, or technical difficulties were experienced during the colonoscopy. Thus, 13 of the original 46 patients completed the entire study; these patients comprised 8 females and 5 males with a mean age of 34 years (range 20–45 years).

### 3.2. Colonoscopies, Histopathology, and Immunohistochemistry

The colon and rectum were macroscopically normal and the histopathological examinations revealed normal structures in both patients and controls. CgA-immunoreactive cells were found in the mucosa of both the colon and rectum of the patients and controls. These cells were either basket- or flask-shaped and sometimes had a long basal cytoplasmic process.

### 3.3. Dietary Assessment

The effect of dietary guidance has been described in detail elsewhere [39]. In brief, the patients had a significantly lower daily total consumption of FODMAPs-rich fruits and vegetables after receiving dietary guidance (9.2 ± 3.2 g) than before receiving dietary guidance (16.2 ± 5.3 g, *P* = 0.016). On the other hand, the daily consumption of dietary fiber did not differ significantly between before (27.4 ± 2.5 g) and after (23.1 ± 2.2 g) receiving dietary guidance (*P* = 0.093).

### 3.4. Computerized Image Analyses

#### 3.4.1. Colon

In the control group, the densities of CgA cells in the total colon, right colon, and left colon were 83.3 ± 10.1, 33.7 ± 5.3, and 49.6 ± 6.0 cells/mm^2^, respectively.

**Figure 1:**
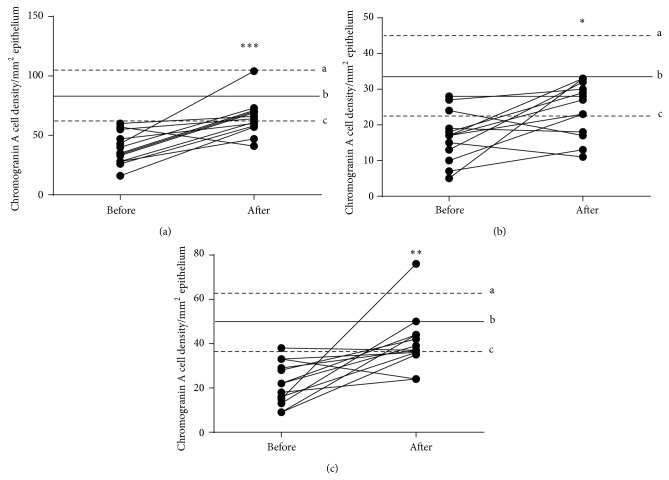
CgA cell densities in the total colon (a), right colon (b), and left colon (c) of IBS patients before and after receiving dietary guidance. The dashed lines labeled “a” and “c” indicate the upper and lower limits of the 95% confidence interval for control subjects, respectively, while line “b” indicates the mean CgA cell density. ^∗^ *P* < 0.05. ^∗∗^ *P* < 0.001. ^∗∗∗^ *P* < 0.0001.

**Figure 2:**
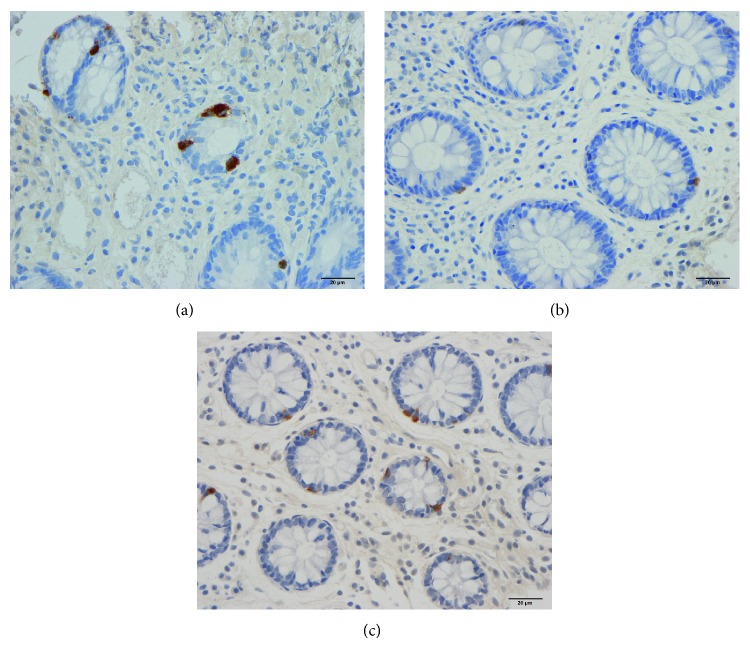
CgA-immunoreactive cells in the total colon of a control subject (a) and of an IBS patient before (b) and after (c) receiving dietary guidance.

The densities of CgA cells in the total colon of IBS patients were 38.6 ± 3.7 and 64.7 ± 4.2 cells/mm^2^ before and after receiving dietary guidance, respectively; this increase was statistically significant (*P* = 0.0004) (Figures 1(a) and 2). The densities of CgA cells before and after receiving dietary guidance were 16.7 ± 1.9 and 24.4 ± 2.1 cells/mm^2^, respectively, in the right colon, and 21.9 ± 2.7 and 40.3 ± 3.6 cells/mm^2^ in the left colon. The increases in CgA cell densities in both the right colon (Figure 1(b)) and left colon (Figure 1(c)) were statistically significant (*P* = 0.0157 and 0.0039, resp.). By comparing CgA cell densities between males and females, no statistically significant difference was found before and after receiving dietary guidance (Table 1).

#### 3.4.2. Rectum

**Figure 3:**
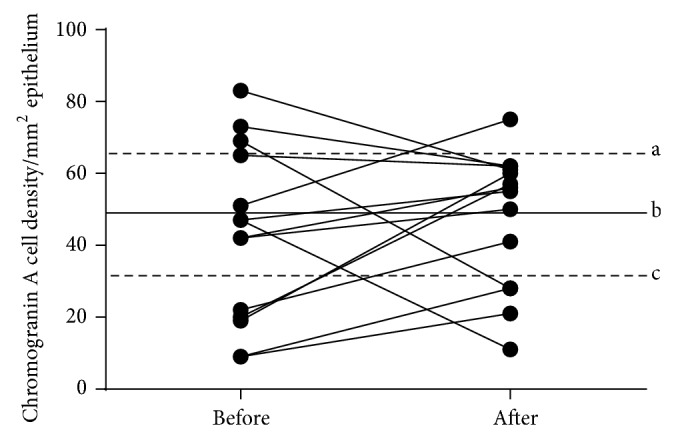
CgA cell density in the rectum of IBS patients before and after receiving dietary guidance. The symbols are the same as in Figure 1.

**Figure 4:**
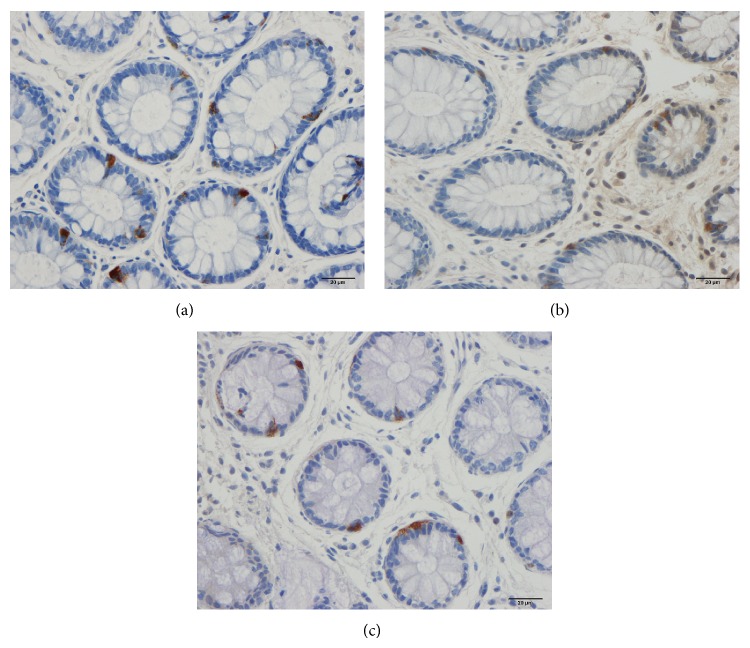
CgA-immunoreactive cells in the rectum of a control subject (a) and of an IBS patient before (b) and after (c) receiving dietary guidance.

The densities of the CgA cells in the rectum were 49.0 ± 7.9, 42.7 ± 6.5, and 47.6 ± 5.0 cells/mm^2^ in the controls and in the IBS patients before and after receiving dietary guidance, respectively (Figures 3 and 4). The cell density in the rectum did not differ significantly between before and after receiving dietary guidance in the IBS patients (*P* = 0.47). In addition, no significant difference was found in CgA cell density between males and females before and after receiving dietary guidance (Table 1).

## 4. Discussion

The dropout rate of patients with IBS is reportedly high in clinical studies, ranging between 33% and 48% [40, 45–48]. The current study included two colonoscopies and needed the patients to follow a strict diet for at least 3 months. This demanding design may have contributed to the even higher dropout rate of 52%. Most of these dropouts were due to improvements in the patients' symptoms after receiving dietary guidance. Additional factors further increased the dropout rate to 72%, such as exclusion after being diagnosed with celiac disease, lupus, pregnancy, moving abroad, and technical difficulties experienced during the colonoscopy.

CgA is a member of the granin (chromogranin-secretogranin) family located within the vesicles of neurons and endocrine cells [30, 31, 49], and it serves as a marker for the GI endocrine cells, endocrine tumors, and gut inflammation [30–33]. A significant change in the densities of the endocrine cells of the colon occurred following changes in the diet in the IBS patients, with the values moving towards those measured for the control subjects. The density of CgA cells in the colon was abnormal in IBS patients before receiving dietary guidance, which is in line with a previous report [33]. However, the density of CgA cells in the rectum of IBS patients did not differ from the control subjects, which was in line with what was reported previously [27]. Such findings were attributed to the physiological functions differing between the colon and rectum, in that the colon absorbs water, sodium, and some fat-soluble vitamins, whereas the rectum's sole role is as a reservoir for the feces prior to defecation [27].

There are contradictory results concerning the blood levels of CgA in IBS. Some results show low levels of serum CgA [50], and others show higher levels of CgA [51] or unchanged levels in IBS patients [33]. As CgA-immunoreactive cells occur in all the segments of the GI tract, the different serum levels reflected segments other than large intestine or stomach.

As described previously, the main triggers of the GI endocrine cells are the luminal contents of the GI tract, especially the nutrients [7, 36]. The turnover from stem cells to mature endocrine cells is rapid [52, 53]. It can be speculated that changing the pattern of food intake—with the subsequent improvement in IBS symptoms—can alter the differentiation of the endocrine cells and explain the observed increase in the CgA cell density in both the colon and rectum towards the values measured for the control subjects. The interaction between food type and food intake is a dynamic process [54].

The CgA cell densities in the colon and the rectum did not differ between males and females before receiving dietary guidance, which is in line with previous results [28], nor was there any difference in the cell densities after receiving dietary guidance, as shown in Table 1.

A previous study has shown that the density of CgA cells is abnormal in IBS patients and was reduced in the colon indicating a reduction in the density of total endocrine cells [27]. These cells interact with intraluminal nutrients in the gut and release different hormones accordingly and control the functions of the GI tract. Since the symptoms of most IBS patients developed after consuming certain foods, one could speculate that there might be a relationship between diet and the GI endocrine cells. The findings of the present study show that the GI endocrine cells may be involved in the pathophysiology of IBS and symptom development and that dietary guidance is an important key factor in managing and improving IBS symptoms. After receiving dietary guidance the densities of these cells normalize as well as the IBS symptoms, which indicates that these cells play a role in the pathogenesis of IBS, providing that they were abnormal under symptom development and normal again when the symptoms improved. The change in the CgA cell density after receiving dietary guidance may reflect a change in the densities of the large intestinal endocrine cells. Future studies should attempt to determine which endocrine cell type(s) is (are) affected.

## Figures and Tables

**Table 1:** Densities of chromogranin A cells in the total colon, right colon, left colon, and rectum of the patients, defined by their gender, before and after receiving dietary guidance.

Location	Endocrine cell densities (cells/mm^2^)				*P* values before guidance	*P* values after guidance
	Males before guidance	Females before guidance	Males after guidance	Females after guidance		
Total colon	37.8	41.1	68.6	65.9	0.7	0.8
Right colon	18.2	17	21.4	28.1	0.8	0.1
Left colon	19.6	23.4	47.2	36	0.5	0.1
Rectum	53.8	36.6	55.8	43.1	0.2	0.2

## References

[1] El-Salhy M., Gundersen D., Hatlebakk J. G., Hausken T. (2012). *Irritable Bowel Syndrome*.

[2] El-Salhy M., Østgaard H., Gundersen D., Hatlebakk J. G., Hausken T. (2012). The role of diet in the pathogenesis and management of irritable bowel syndrome (review). *International Journal of Molecular Medicine*.

[3] Agreus L., Svardsudd K., Nyren O., Tibblin G. (1995). Irritable bowel syndrome and dyspepsia in the general population: overlap and lack of stability over time. *Gastroenterology*.

[4] Drossman D. A., Li Z., Andruzzi E. (1993). U. S. Householder survey of functional gastrointestinal disorders—Prevalence, sociodemography, and health impact. *Digestive Diseases and Sciences*.

[5] El-Salhy M. (2012). Irritable bowel syndrome: diagnosis and pathogenesis. *World Journal of Gastroenterology*.

[6] Thompson W. G., Heaton K. W. (1980). Functional bowel disorders in apparently healthy people. *Gastroenterology*.

[7] El-Salhy M., Gundersen D., Gilja O. H., Hatlebakk J. G., Hausken T. (2014). Is irritable bowel syndrome an organic disorder?. *World Journal of Gastroenterology*.

[8] Lee Y. J., Park K. S. (2014). Irritable bowel syndrome: emerging paradigm in pathophysiology. *World Journal of Gastroenterology*.

[9] Camilleri M. (2014). Physiological underpinnings of irritable bowel syndrome: neurohormonal mechanisms. *The Journal of Physiology*.

[10] El-Salhy M. (2002). The possible role of the gut neuroendocrine system in diabetes gastroenteropathy. *Histology and Histopathology*.

[11] Moran G. W., Leslie F. C., Levison S. E., Worthington J., McLaughlin J. T. (2008). Enteroendocrine cells: neglected players in gastrointestinal disorders?. *Therapeutic Advances in Gastroenterology*.

[12] Moran-Ramos S., Tovar A. R., Torres N. (2012). Diet: friend or foe of enteroendocrine cells: how it interacts with enteroendocrine cells. *Advances in Nutrition*.

[13] Buffa R., Capella C., Fontana P., Usellini L., Solcia E. (1978). Types of endocrine cells in the human colon and rectum. *Cell and Tissue Research*.

[14] Sandström O., El-Salhy M. (1999). Ageing and endocrine cells of human duodenum. *Mechanisms of Ageing and Development*.

[15] El-Salhy M. (2009). Ghrelin in gastrointestinal diseases and disorders: a possible role in the pathophysiology and clinical implications (review). *International Journal of Molecular Medicine*.

[16] Tolhurst G., Reimann F., Gribble F. M. (2012). Intestinal sensing of nutrients. *Handbook of Experimental Pharmacology*.

[17] Lee J., Cummings B. P., Martin E. (2012). Glucose sensing by gut endocrine cells and activation of the vagal afferent pathway is impaired in a rodent model of type 2 diabetes mellitus. *American Journal of Physiology—Regulatory Integrative and Comparative Physiology*.

[18] Parker H. E., Reimann F., Gribble F. M. (2010). Molecular mechanisms underlying nutrient-stimulated incretin secretion. *Expert Reviews in Molecular Medicine*.

[19] Raybould H. E. (2008). Nutrient sensing in the gastrointestinal tract: possible role for nutrient transporters. *Journal of Physiology and Biochemistry*.

[20] san Gabriel A., Nakamura E., Uneyama H., Torii K. (2009). Taste, visceral information and exocrine reflexes with glutamate through umami receptors. *The Journal of Medical Investigation*.

[21] Rudholm T., Wallin B., Theodorsson E., Näslund E., Hellström P. M. (2009). Release of regulatory gut peptides somatostatin, neurotensin and vasoactive intestinal peptide by acid and hyperosmolal solutions in the intestine in conscious rats. *Regulatory Peptides*.

[22] Sternini C., Anselmi L., Rozengurt E. (2008). Enteroendocrine cells: a site of ‘taste’ in gastrointestinal chemosensing. *Current Opinion in Endocrinology, Diabetes and Obesity*.

[23] Sternini C. (2007). Taste receptors in the gastrointestinal tract. IV. Functional implications of bitter taste receptors in gastrointestinal chemosensing. *American Journal of Physiology—Gastrointestinal and Liver Physiology*.

[24] Buchan A. M. J. (1999). Nutrient tasting and signaling mechanisms in the gut III. Endocrine cell recognition of luminal nutrients. *American Journal of Physiology—Gastrointestinal and Liver Physiology*.

[25] Montero-Hadjadje M., Elias S., Chevalier L. (2009). Chromogranin A promotes peptide hormone sorting to mobile granules in constitutively and regulated secreting cells. Role of conserved N- and C-terminal peptides. *The Journal of Biological Chemistry*.

[26] Shooshtarizadeh P., Zhang D., Chich J.-F. (2010). The antimicrobial peptides derived from chromogranin/secretogranin family, new actors of innate immunity. *Regulatory Peptides*.

[27] El-Salhy M., Mazzawi T., Gundersen D., Hausken T. (2012). Chromogranin A cell density in the rectum of patients with irritable bowel syndrome. *Molecular Medicine Reports*.

[28] Sandström O., El-Salhy M. (1999). Human rectal endocrine cells and aging. *Mechanisms of Ageing and Development*.

[29] Sjolund K., Sanden G., Hakanson R., Sundler F. (1983). Endocrine cells in human intestine: an immunocytochemical study. *Gastroenterology*.

[30] Taupenot L., Harper K. L., O'Connor D. T. (2003). The chromogranin-secretogranin family. *The New England Journal of Medicine*.

[31] Wiedenmann B., Huttner W. B. (1989). Synaptophysin and chromogranins/secretogranins—widespread constituents of distinct types of neuroendocrine vesicles and new tools in tumor diagnosis. *Virchows Archiv B: Cell Pathology Including Molecular Pathology*.

[32] Deftos L. J. (1991). Chromogranin A: its role in endocrine function and as an endocrine and neuroendocrine tumor marker. *Endocrine Reviews*.

[33] El-Salhy M., Lomholt-Beck B., Hausken T. (2010). Chromogranin a as a possible tool in the diagnosis of irritable bowel syndrome. *Scandinavian Journal of Gastroenterology*.

[34] El-Salhy M., Hatlebakk J. G., Gilja O. H., Hausken T. (2014). Irritable bowel syndrome: recent developments in diagnosis, pathophysiology, and treatment. *Expert Review of Gastroenterology & Hepatology*.

[35] El-Salhy M. (2014). Endocrine cells in the oxyntic mucosa of the stomach in patients with irritable bowel syndrome. *World Journal of Gastrointestinal Endoscopy*.

[36] El-Salhy M., Hatlebakk J. G., Gilja O. H., Hausken T. (2014). Irritable bowel syndrome: recent developments in diagnosis, pathophysiology, and treatment. *Expert Review of Gastroenterology and Hepatology*.

[37] Simrén M., Månsson A., Langkilde A. M. (2001). Food-related gastrointestinal symptoms in the irritable bowel syndrome. *Digestion*.

[38] Williams E. A., Nai X., Corfe B. M. (2011). Dietary intakes in people with irritable bowel syndrome. *BMC Gastroenterology*.

[39] Mazzawi T., Hausken T., Gundersen D., Magdy E.-S. (2013). Effects of dietary guidance on the symptoms, quality of life and habitual dietary intake of patients with irritable bowel syndrome. *Molecular Medicine Reports*.

[40] Østgaard H., Hausken T., Gundersen D., El-Salhy M. (2012). Diet and effects of diet management on quality of life and symptoms in patients with irritable bowel syndrome. *Molecular Medicine Reports*.

[41] Mazzawi T., Gundersen D., Hausken T., El-Salhy M. (2014). Increased gastric chromogranin A cell density following changes to diets of patients with irritable bowel syndrome. *Molecular Medicine Reports*.

[42] Mazzawi T., Hausken T., Gundersen D., El-Salhy M. (2014). Effect of dietary management on the gastric endocrine cells in patients with irritable bowel syndrome. *European Journal of Clinical Nutrition*.

[43] Masson L. F., McNeill G., Tomany J. O. (2003). Statistical approaches for assessing the relative validity of a food-frequency questionnaire: use of correlation coefficients and the kappa statistic. *Public Health Nutrition*.

[44] Brantsaeter A. L., Haugen M., Alexander J., Meltzer H. M. (2008). Validity of a new food frequency questionnaire for pregnant women in the Norwegian Mother and Child Cohort Study (MoBa). *Maternal and Child Nutrition*.

[45] Enck P., Klosterhalfen S., Kruis W. (2005). Determination of placebo effect in irritable bowel syndrome. *Deutsche Medizinische Wochenschrift*.

[46] Abdul-Baki H., El Hajj I. I., Elzahabi L. (2009). A randomized controlled trial of imipramine in patients with irritable bowel syndrome. *World Journal of Gastroenterology*.

[47] Zernicke K. A., Campbell T. S., Blustein P. K. (2013). Mindfulness-based stress reduction for the treatment of irritable bowel syndrome symptoms: a randomized wait-list controlled trial. *International Journal of Behavioral Medicine*.

[48] Halmos E. P., Power V. A., Shepherd S. J., Gibson P. R., Muir J. G. (2014). A diet low in FODMAPs reduces symptoms of irritable bowel syndrome. *Gastroenterology*.

[49] Khan W. I., Ghia J. E. (2010). Gut hormones: emerging role in immune activation and inflammation. *Clinical & Experimental Immunology*.

[50] Valeur J., Milde A. M., Helle K. B., Berstad A. (2008). Low serum chromogranin a in patients with self-reported food hypersensitivity. *Scandinavian Journal of Gastroenterology*.

[51] Helle K. B., Corti A., Metz-Boutigue M.-H., Tota B. (2007). The endocrine role for chromogranin A: a prohormone for peptides with regulatory properties. *Cellular and Molecular Life Sciences*.

[52] Hocker M., Wiedenmann B. (1998). Molecular mechanisms of enteroendocrine differentiation. *Annals of the New York Academy of Sciences*.

[53] Inokuchi H., Fujimoto S., Kawai K. (1983). Cellular kinetics of gastrointestinal mucosa, with special reference to gut endocrine cells. *Archivum Histologicum Japonicum*.

[54] El-Salhy M., Gilja O. H., Gundersen D., Hatlebakk J. G., Hausken T. (2014). Interaction between ingested nutrients and gut endocrine cells in patients with irritable bowel syndrome (review). *International Journal of Molecular Medicine*.

